# Crocin Prevents Sub-Cellular Organelle Damage, Proteolysis and Apoptosis in Rat Hepatocytes: A Justification for Its Hepatoprotection

**Published:** 2018

**Authors:** Bahareh Sadat Yousefsani, Soghra Mehri, Jalal Pourahmad, Hossein Hosseinzadeh

**Affiliations:** a *Department of Pharmacodynamy and Toxicology, School of Pharmacy, Mashhad University of Medical Sciences, Mashhad, Iran. *; b *Pharmaceutical Research Center, Pharmaceutical Technology Institute, Mashhad University of Medical Sciences, Mashhad, Iran.*; c *Department of Pharmacology and Toxicology, Faculty of Pharmacy, Shahid Beheshti University of Medical Sciences, Tehran, Iran.*

**Keywords:** Crocin, Hepatoprotection, Oxidative stress, Isolated rat hepatocyte, Antioxidant

## Abstract

Crocin, the main constituent of saffron (*Crocus sativus* L.), is a natural carotenoid which is known for its antioxidant activity. Liver as the organ that metabolizes many chemicals is one of the first position that is at risk of environmental pollutants. It is clear that compounds that exhibit antioxidant properties, scavenging of free radicals and inhibition of lipid peroxidation are expected to show hepatoprotective effects. Previous studies have proven the protective effect of crocin on the liver. The aim of this study is to find out the exact hepatoprotective mechanisms of this compound. In the present study, the protective effects of various concentrations of crocin (5, 10, 25, 50 and 100 μg/mL) were examined against oxidative stress toxicity induced by cumene hydroperoxide (CHP) on isolated rat hepatocytes. To find out the exact protective activity of crocin, we evaluated cell lysis, lipid peroxidation, reactive oxygen species (ROS) generation, GSH/GSSG, collapse of mitochondrial membrane potential, lysosomal membrane damage, the release of cytochrome c, and cellular proteolysis. Crocin (50 and 100 µg/mL) reduces cell lysis, lipid peroxidation, ROS generation, collapse of mitochondrial membrane potential, lysosomal membrane damage, cytochrome c release, and cellular proteolysis. It also increase GSH/GSSG. Crocin (50 and 100 µg/mL) reduced liver toxicity not only as an antioxidant but also by protecting the mitochondria and lysosome. Our data demonstrated that crocin is a promising candidate for preventing liver injury associated with oxidative stress. These findings pave the way to further studies evaluating the clinical protective effect of crocin.

## Introduction

Saffron (*Crocus sativus*, L.) has been used as an herbal food additive as well as a flavoring and coloring factor. Crocin, crocetin, and safranal are the main chemical constituents of saffron. The color of saffron is due to the presence of crocin, which have a glycoside carotenoid structure (1). Many organs of the body are affected by pharmacological activities of crocin, for example, anticonvulsant, antidepressant and neuroprotective effect on nervous system (2), antiatherosclerosis and antihyperlipidemic effect on cardiovascular system (3) and beneficial effect on sexual behavior in reproductive system (4). Crocin protected liver against acute damages (5). Crocin has a reducing effect on inflammation (6). Many studies have illustrated the antiradical scavenging efficacy of crocin (7). It seems that neuroprotective, anti-aging, anti-inflammatory, and antitumor activities of crocin arises out of its antioxidant effect (8). Soeda *et al.*, Showed the efficacy of crocin at inhibition of oxidative stress-induced cell death is due to a GSH-dependent mechanism (9). As it is clear crocin is a potent antioxidant (10). As we know, one of the major causes of initiation and progression of liver disease is oxidative stress. This is a feature commonly observed in a wide range of liver diseases, including alcoholic or nonalcoholic steatohepatitis and viral hepatitis (11). Liver as the major site that metabolizes many xenobiotics can be injured by exposure to various toxic chemicals, drugs, infection, *etc.* (12). Many plants rooted from the traditional medicine are endowed with hepatoprotective properties (13). In one study, crocin at 0.1% in the diet, prevented ratʹs hepatic injury induced by aflatoxin B1 and dimethylnitrosamine through suppressing serum levels of enzymes like alkaline phosphatase and lactate dehydrogenase (LDH) (14). In another study, crocin protected liver against nicotine-induced damages (5). Due to the long records of antioxidant and free radical scavenging properties of crocin, we planned to study the hepatoprotective effects of this compound against various cellular and sub-cellular characteristics. Cell lysis, lipid peroxidation, reactive oxygen species (ROS) generation, GSH/GSSG, collapse of mitochondrial membrane potential, lysosomal membrane damage, and cellular proteolysis were evaluated to find out the exact hepatoprotective mechanism of crocin. We used isolated Sprague–Dawley rat hepatocytes as cellular model. Isolated hepatocyte cells are the most similar mammalian cells to human liver hepatocytes (15).

## Experimental

*Quantification of crocin*

A rectified method was used to quantify crocin in an aqueous saffron extract (16). The purity of crocin was 95%.

*Chemicals*

Rhodamine 123, collagenase, bovine serum albumin (BSA), N-(2-hydroxyethyl) piperazine-N0-(2-ethanesulfonic acid) (HEPES), o-phthaldialdehyde (OPA), reduced and oxidized glutathione (GSH and GSSG), acridine orange, 20,70-dichlorofluorescin diacetate (DCFHDA), trichloroacetic acid, trypan blue, heparin and cumene hydroperoxide were purchased from Sigma–Aldrich Co. Sodium dodecyl sulfate (SDS) was purchased from Bio-Rad Laboratories (Germany). 

*Animals*

Male Sprague-Dawley rats weighing 280 to 300 g were used in the study. All rats were housed in a room at a constant temperature of 25 °C on a 12/12 h light/dark cycle with food and water available ad libitum. All experiments were handled according to ethical standards and protocols approved by the Committee of Animal Experimentation of Mashhad University of Medical Sciences, Mashhad, Iran.

*Isolation and incubation of hepatocytes*

Taking the hepatocytes were done by collagenase perfusion of the liver and viability was analyzed by trypan blue staining method (17). 

*Cell viability*

The survival of isolated hepatocytes was performed with the intactness of the plasma membrane as determined by the trypan blue exclusion test (17). Taking the samples of the incubated hepatocyte was done at different time points during the 3 h incubation time. The control cells were still alive after 3 h in the percentage of 80–90% (18).

*Crocin treatment*

Hepatocytes were exposed to crocin 30 min before a toxic compound *i.e.* cumene hydroperoxide (CHP), to prevent hepatotoxicity. CHP (EC50 = 120 µM (13)) has been frequently used as a model compound for organic hydroperoxides and for the study of mechanisms of oxidative cell injury in mammalian cells (13). A wide range of crocin concentration (1-100 mg/mL) was used in our pilot study and their inhibitory effects against CHP induced hepatocyte toxicity were evaluated 

(2).

*Determination of reactive oxygen species*

To determine the rates of hepatocyte ROS formation induced by CHP, dichlorofluorescindiacetate (1.6 μM) was added to the hepatocytes containing flasks. ROS were determined spectrofluorometrically by the measurement of highly florescent DCF. The results were expressed as fluorescent intensity per 10^6^ cells (19).

*Lipid peroxidation assay*

Hepatocyte lipid peroxidation was measured by measuring the amount of thiobarbituric acid-reactive substances (TBARS) formed during the decomposition of lipid hydroperoxides. The absorbance measured spectrophotometerically (20). Gallic acid was used as a positive control in this test.

*Mitochondrial membrane potential (MMP) assay*

Mitochondrial uptake of the cationic fluorescent dye, rhodamine123, has been used for estimation of mitochondrial membrane potential (23). Our data were shown as the percentage of MMP collapse (%ΔΨm) in all treated (test) hepatocyte group. Carnitine is used as a positive control in this test.

*Lysosomal membrane integrity assay*

Hepatocyte lysosomal membrane stability was evaluated by redistribution of the fluorescent dye, acridine orange (24). Our data were shown as the percentage of lysosomal membrane leakiness in all treated (test) hepatocyte groups. Cholorquine was used as a positive control in this test.

*Intracellular GSH and extra cellular GSSG assessment*

GSH and GSSG were evaluated according to the spectrofluorometric method (21). In this method a reaction between orthophetaldehyde (OPA) and GSH (in pH 8) and GSSG (in pH 12) formed a fluorescent substance. Oxidized and reduced glutation were positive controls used in this test.

*Measurement of proteolysis*

Proteolysis was determined with spectrophotometer assay using Church *et al.* method (24). For using this method at first an aliquot of cell suspension was precipitated with an equal volume of 20% trichloroacetic acetic acid and allowed to stand 12 h at 4 ºC. Then, o-phthaldialdehyde (OPA) solution reagent was prepared for determination of proteolysis. This method is based on the reaction of OPA and β-mercaptoethanol with primary amines. The determination of proteolysis was carried out by spectrophotometer set at 340 nm. Gallic acid was used as a positive control in this test.

*Statistical analysis*

The statistical analysis was done using the Graph Pad Prism software, version 6 (Graph Pad Software, San Diego, CA, USA). Data are reported as mean ± standard deviation (SD) of tree separated tests. The data were analyzed using one-way analysis of variance (ANOVA) followed by Tukey-Kramer test as the post hoc test. The minimal level of significance chosen was *P* < 0.05. 

## Results and Discussion

*Cell cytotoxicity*

**Figure 1:**
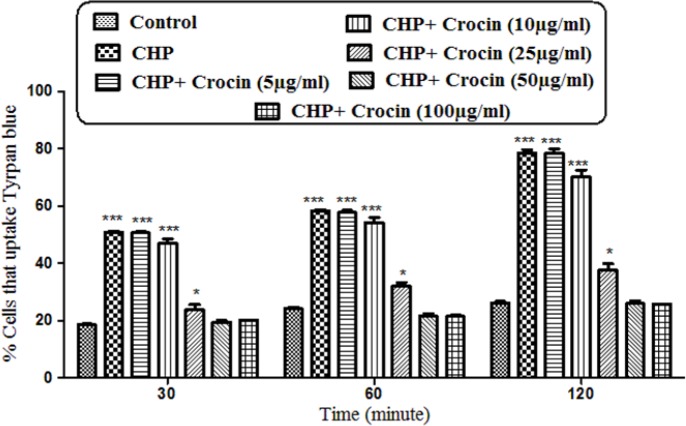
Preventing effect of different concentrations of crocin against CHP induced hepatocyte lysis. Isolated rat hepatocytes at the concentration of 10^6 ^cells/mL incubated in Krebs–Henseleit buffer pH 7.4 at 37 ºC. Determination of cytotoxicity was done as the percentage of cells that absorb trypan blue. (CHP: cumene hydroperoxide) values are shown as mean ± SD of three separate experiments (n = 3). *P < 0. 05, ***P < 0.001, significant difference in comparison with non-treated hepatocytes (control). ###*P* < 0.001 significant difference in comparison with CHP treated hepatocyte

As shown in Figure 1 CHP induced cytotoxicity 30, 60, and 120 min after incubation. Pretreatment of cells with crocin (50 and 100 µg/mL) significantly (*P* < 0.05) prevented CHP induced hepatocyte membrane lysis. There was no significant difference between treatment with crocin at concentrations of 50 and 100 µg/mL.

**Figure 2:**
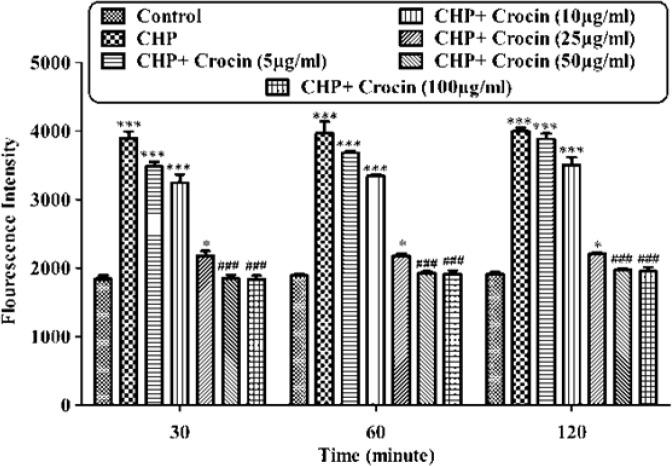
Preventing CHP induced intracellular ROS formation by different concentrations of crocin. Isolated rat hepatocytes at the concentration of 10^6^ cells/mL were incubated in Krebs–Henseleit buffer (pH 7.4) at 37 ºC. Reactive oxygen specious (ROS) were determined spectrofluorometrically by the measurement of highly florescent DCF. (CHP: cumene hydroperoxide), values are shown as mean ± SD of three separate experiments (n = 3). *P < 0.05, ***P < 0.001, significant difference in comparison with non-treated hepatocytes (control). ###*P* < 0.001 significant difference in comparison with CHP treated hepatocyte

**Figure 3:**
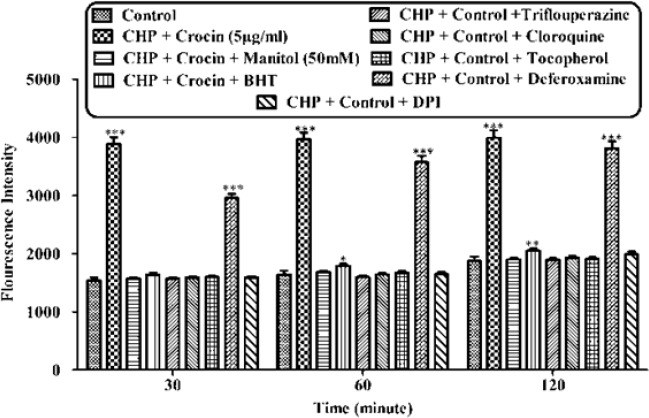
Effect of concurrent use of non-effective concentration of crocin (5 µg/mL) and lipid antioxidant, MPT pore sealing agents, ferric chelator, ROS scavengers, lysosomotropic against CHP-induced hepatocyte lysis, against CHP induced ROS formation on isolated rat hepatocytes. Isolated rat hepatocytes at the concentration of 10^6^ cells/mL were incubated in Krebs–Henseleit buffer (pH 7.4) at 37 ºC. Reactive oxygen specious (ROS) were determined spectrofluorometrically by the measurement of highly florescent DCF. (CHP: cumene hydroperoxide) values are shown as mean ± SD of three separate experiments (n = 3). *P < 0.05, **P < 0.01, ***P < 0.001, significant difference in comparison with non-treated hepatocytes (control

**Figure 4:**
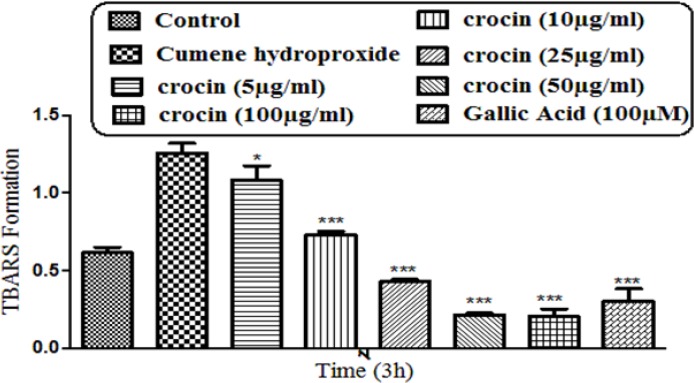
Preventing CHP induced lipid peroxidation by different concentrations of crocin and gallic acid (100 µM). Isolated rat hepatocytes at the concentration of 10^6^ cells/mL were incubated in Krebs–Henseleit buffer (pH 7.4) at 37 ºC. TBARS formation was measured spectrophotometrically and expressed as µM concentrations. (CHP: cumene hydroperoxide) values are shown as mean ± SD of three separate experiments (n = 3). **P < 0.01, ***P < 0.001, significant difference in comparison with non-treated hepatocytes (control). ###*P* < 0.001 significant difference in comparison with CHP treated hepatocyte

**Figure 5:**
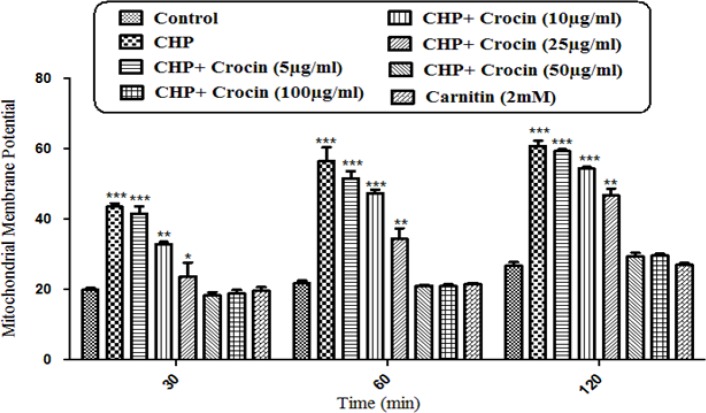
Preventing CHP induced mitochondrial membrane potential collapse by different concentrations of crocin and carnitine. Isolated rat hepatocytes at the concentration of 10^6 ^cells/mL incubated in Krebs–Henseleit buffer pH 7.4 at 37 ºC. The difference in mitochondrial uptake of the rhodamine 123 between the untreated control and CHP treated cells is the biochemical basis for the measurement of the percentage of mitochondrial membrane potential decline. Our data showed significant (*P* < 0.05) decrease in mitochondrial membrane potential collapse (%ΔΨm) by crocin at concentrations of 50 and 100 mg/mL, but the concentration of 50 µg/mL was better than other concentrations. (CHP: cumene hydroperoxide) values are shown as mean ± SD of three separate experiments (n = 3). *P < 0.05, ***P < 0.001, significant difference in comparison with non-treated hepatocytes (control). ###*P* < 0.001 significant difference in comparison with CHP treated hepatocyte

**Figure 6:**
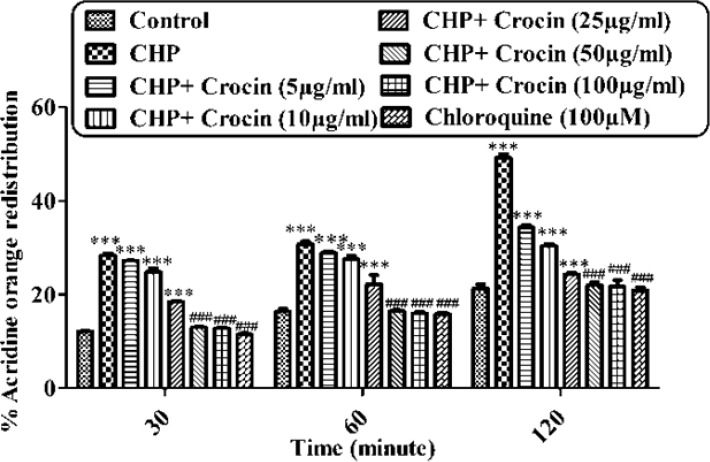
Preventing CHP induced lysosomal membrane injury by different concentrations of crocin. Isolated rat hepatocytes at the concentration of 10^6 ^cells/ml incubated in Krebs–Henseleit buffer pH 7.4 at 37 ºC. The redistribution of acridine orange from lysosomes into cytosol in acridine orange loaded hepatocytes was assigned as a biochemical basis for the measurement of lysosomal membrane injury. Highly florescent acridine orange redistribution was determined spectrofluorometrically in treated hepatocytes and shown as the percentage of hepatocytes lysosomal membrane leakage in all groups in three different time intervals (30, 60 and 120 min). (CHP: cumene hydroperoxide) values are shown as mean ± SD of three separate experiments (n = 3). ***P < 0.001, significant difference in comparison with non-treated hepatocytes (control). ###*P* < 0.001 significant difference in comparison with CHP treated hepatocyte

**Figure 7:**
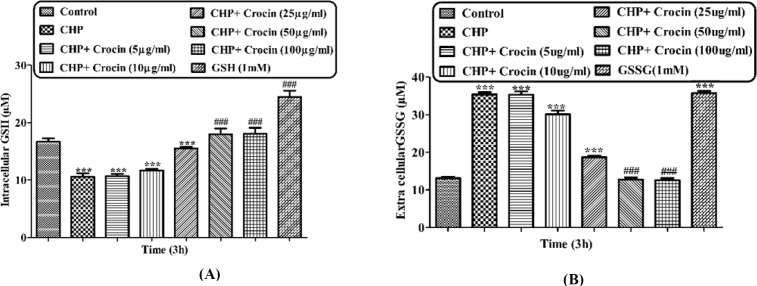
Preventing CHP induced GSH depletion by different concentrations of crocin and net values of (A) GSH and (B) GSSG. Isolated rat hepatocytes at the concentration of 10^6 ^cells/mL incubated in Krebs–Henseleit buffer pH 7.4 at 37 ºC. Intracellular GSH and extra cellular GSSG were measured spectrofluorometrically. (CHP: cumene hydroperoxide) values are shown as mean ± SD of three separate experiments (n = 3). *P < 0.05, **P < 0.01, ***P < 0.001, significant difference in comparison with non-treated hepatocytes (control). ###*P* < 0.001 significant difference in comparison with CHP treated hepatocyte

**Figure 8:**
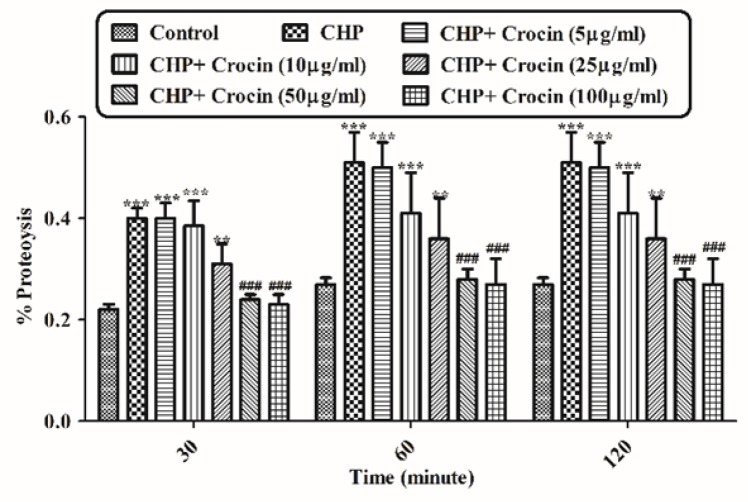
Preventing CHP induced proteolysis by different concentrations of crocin. Isolated rat hepatocytes at the concentration of 10^6 ^cells/mL incubated in Krebs–Henseleit buffer pH 7.4 at 37 ºC. Determination of proteolysis was carried out spectrophotometrically using wavelength ( max) 340 nm. Data was shown as the percentage of proteolysis in comparison with control group. (CHP: cumene hydroperoxide) values are shown as mean ± SD of three separate experiments (n = 3). **P < 0.01, ***P < 0.001, significant difference in comparison with non-treated hepatocytes (control). ###*P* < 0.001 significant difference in comparison with CHP treated hepatocyte

*Determination of reactive oxygen species*

As shown in Figure 2, CHP induced ROS generation. Crocin at concentrations of 50 and 100 µg/mL significantly (*P* < 0.05) prevented CHP induced ROS formation but there was no significant difference between treatment with crocin at concentrations of 50 and 100 µg/mL. Lower concentration of crocin (5 µg/mL) did not induce any ROS formation following 120 min of incubation. As shown in Figure 3, CHP-induced ROS generation was prevented by lipid antioxidant (α-Tocopherol succinate), MPT pore sealing agents (carnitine, trifluoperazine), hydroxyl radical scavengers (mannitol), ferric chelator (deferoxamine), lysosomotropic agents (chloroquine), and NADPH P450 reductase inhibitor (diphenyliodonium chloride). All of these protective agents did not have any toxic effect on hepatocytes at concentrations which were used. When each of these inhibitors was applied simultaneously with non-effective concentration of crocin (5 µg/mL), much better inhibitory effect was seen against CHP induced ROS formation than the inhibitor alone (data not shown).

*Lipid peroxidation assay*

As demonstrated in Figure 4, CHP induced a significant (*P* < 0. 05) increase at TBARS levels within 120 min of incubation in isolated rat hepatocytes. Crocin at concentrations of 25, 50, and 100 mg/mL significantly (*P* < 0.05) prevented CHP induced lipid peroxidation, but there was no significant difference between treatment with crocin at concentrations of 50 and 100 µg/mL.

*Mitochondrial membrane potential (MMP) assay*

As a result of hepatocyte ROS formation, CHP induced a rapid decline of mitochondrial membrane potential, an apparent marker of mitochondrial dysfunction. MMP decrease was significantly (*P* < 0.05) prevented by crocin at concentrations of 50 and 100 mg/mL, but there was no significant difference between treatment with crocin at concentrations of 50 and 100 µg/mL (Figure 5).

*Lysosomal membrane completeness assay*

When the lysosomes of hepatocyte were loaded with a lysosomotropic agent, sever oxidative damage to lysosomal membrane was occurred following the incubation of CHP. CHP demonstrated a significant release of acridine orange into the cytosolic fraction ensued within 120 min of incubation with 120 µM (Figure 6). CHP induced acridine orange release was prevented by crocin at concentrations of 50 and 100 µg/mL significantly (*P* < 0.05), but there was no significant difference between treatment with crocin at concentrations of 50 and 100 µg/mL.

*Intracellular GSH and extra cellular GSSG evaluation*

Incubation of hepatocytes with CHP caused rapid hepatocyte glutathione (GSH) depletion, another marker of cellular oxidative stress (Figure 7A). Most of the CHP induced glutathione depletion could lead to produce GSSG (Figure 7B). We therefore, determined the glutathione depletion in CHP treated and control hepatocytes by measuring decrease of intracellular GSH and increase of extra cellular GSSG. CHP induced glutathione depletion was significantly (*P* < 0.05) prevented by different concentrations of crocin (50 and 100 mg/mL and crocin at 50 µg/mL prevented much better than other concentrations.

*Measurement of proteolysis*

Hepatocyte proteolysis occurred due to lysosomal membrane damage and release of lysosomal proteases following exposure to CHP (120 µM). Therefore, protein breaks down into amines. In this experiment o-phthaldialdehyde (OPA) and β-mercaptoethanol reacted with primary amines and the absorbance at 340 nm was measured. Increasing the absorption rate, indicating an increase of amines, result in increased proteolysis. As shown in Figure 8, CHP induced proteolysis was significantly (*P* < 0.05) prevented by different concentrations of crocin (50 and 100 µg/mL) and it was no significant difference between treatment with crocin at concentrations of 50 and 100 µg/mL. Data were shown as the percentage of proteolysis in comparison with control group.

Although the protective effect of crocin as an antioxidant has been studied extensively (10), there are only a few data on the protective use of crocin on liver damage. As the major site of xenobiotic metabolism, liver can be influenced by many medicines, chemicals, and infections (11). In the current study, the antioxidant activity of crocin was evaluated in some *in-vitro* experimental models and we tried to find out whether oxidative stress induced cytotoxicity in hepatocytes could be diminished by different concentrations of this natural carotenoid. Our results demonstrated that when isolated hepatocytes were incubated with CHP, there was an initial rapid increase in ROS formation and cell death, and crocin at the concentrations of 50 and 100 µM prevented CHP induced hepatocyte ROS formation (Figures 1 and 2). Previously, it was shown that ROS formation in hepatocyte was prevented by hydroxyl radical scavengers such as dimethyl sulfoxide and mannitol (25). As shown in Figure 3, CHP-induced ROS generation was also prevented by lipid antioxidant (α-Tocopherol succinate), hydroxyl radical scavengers (mannitol), MPT pore sealing agents (carnitine, trifluoperazine), lysosomotropic agents (chloroquine), and NADPH P450 reductase inhibitor (diphenyliodonium chloride) but not with ferric chelator (deferoxamine). All of these protective agents did not show any toxic effects on hepatocytes at concentrations which are used. When these inhibitors/antagonists incubated simultaneously with a non-effective dose of crocin (5 µg/mL), each of them demonstrated better effect compared to their sole treatment (data not shown). We therefore, concluded that crocin could reduce oxidative stress induced toxicity through different cellular pathways. So, based on previous testing, we predicted some of crocin antioxidant mechanisms and following them, further experiments were designed. One of the most extensively investigated processes induced by free radicals is lipid peroxidation. This process and subsequent sub organelle (mitochondria/lysosomes) membrane damage are important mechanisms underlying the toxicity of several oxidative xenobiotics (26). As reported in previous studies, CHP induced microsomal lipid peroxidation is a common model for evaluating the effectiveness of antioxidants (27). In this study, lipid peroxidation and all the sub-cellular subsequent events induced by CHP were significantly inhibited by crocinat the concentration of 50 and 100 µM. To prove the antioxidant effect of crocin, gallic acid 100 µM (an antioxidant reference standard) was used. According to Figure 4, crocin showed significantly better antioxidant protective effect than gallic acid. As we know both mitochondria and lysosomes are target of ROS. Our results showed (Figures 5 and 6) that both the decline in mitochondrial membrane potential and lysosomal membrane leakiness were occurred in isolated hepatocytes incubated with CHP. Therefore, it seems that this damage to mitochondrial and lysosomal membranes could be a lipid peroxidative consequence of ROS formation. As shown in Figures 5 and 6 the protective effect of crocin was compared with carnitin (a MPT pore sealing agents) and chloroquine (a lysosomotropic agent). Results demonstrated that crocin at the concentration of 50 and 100 µM has the same protective effect on mitochondrial and lysosomal membrane. Glutathione (GSH) as an intracellular antioxidant protects the cell against intracellular ROS formation and lipid peroxidation. The harmful effect of oxidative stress prevented by glutathione either directly or as a substrate of glutathione peroxidases. Oxidized glutathione (GSSG) has generated by these reactions (28). Therefore, glutathione depletion is an important sign of cellular oxidative stress and could be ascribed by the expulsion of GSSG through hepatocytes sinusoidal and canalicular membranes. Our results exhibited that, when isolated hepatocytes were incubated with CHP, glutathione decreased as a result of ROS formation and lipid peroxidation. Oxidative stress cytotoxicity has been increased because of glutathione depletion and lysosomal membrane leakage (15). In Our study crocin at the concentrations of 50 and 100 µg/mL significantly increased the amount of intracellular GSH and decreased the amount of extracellular GSSG. The effects of crocin on cellular GSH may be attributed to its direct antioxidative effects or intensifying biosynthesis of GSH (29). In all of our results there is no significant deference between protective effect of crocin (50 and 100 µg/mL) and gallic acid. Protein degradation and proteolysis occures as another result of ROS formation and lysosomal membrane damage induced by CHP. Crocin at the concentration of 50 and 100 µg/mL by maitaining lysosomal membran stability prevented protolysis almost as same as gallic acid. It is proven that both corruption of mitochondrial membrane potential and cytochrome c release are the important indicators of cell apoptosis and also important endpoints for the determination of mitochondrial dysfunction (30). Our results showed that CHP caused significant expulsion of cytochrome c from mitochondria. Moreover, crocin (50 and 100 µM) pretreatment completely blocked the CHP-induced release of cytochrome c from the mitochondria which supports the hypothesis that the apoptosis induction via CHP is due to an oxidative stress and depends on the opening of the MPT pore.

## Conclusion

Based on the above findings, crocin is a potent antioxidant that has the ability to decrease lipid peroxidation, mitochondrial, and lysosomal membrane damages similar to protective agents (*e.g.* gallic acid, carnitine, and clorquine). It can also increase the intracellular antioxidant (GSH) and decrease extracellular reduced and oxidized glutathione. Protein degradation was also reduced by crocin. Therefore, we conclude that the hepatoprotective effects of crocin is not only through an antioxidant effect, but also other mechanisms were involved. So, it seems that pretreatment by crocin could certainly prevent hepatotoxicity, but further studies are required for human consumption. 
